# Consistency in Motion Event Encoding Across Languages

**DOI:** 10.3389/fpsyg.2021.625153

**Published:** 2021-03-30

**Authors:** Guillermo Montero-Melis

**Affiliations:** ^1^Department of Linguistics, Stockholm University, Stockholm, Sweden; ^2^Max Planck Institute for Psycholinguistics, Nijmegen, Netherlands; ^3^Centre for Research on Bilingualism, Department of Swedish Language and Multilingualism, Stockholm University, Stockholm, Sweden

**Keywords:** event conceptualization, motion events, speaker variability, entropy, cross-linguistic, entrenchment

## Abstract

Syntactic templates serve as schemas, allowing speakers to describe complex events in a systematic fashion. Motion events have long served as a prime example of how different languages favor different syntactic frames, in turn biasing their speakers toward different event conceptualizations. However, there is also variability in how motion events are syntactically framed within languages. Here, we measure the consistency in event encoding in two languages, Spanish and Swedish. We test a dominant account in the literature, namely that variability within a language can be explained by specific properties of the events. This event-properties account predicts that descriptions of one and the same event should be consistent within a language, even in languages where there is overall variability in the use of syntactic frames. Spanish and Swedish speakers (*N* = 84) described 32 caused motion events. While the most frequent syntactic framing in each language was as expected based on typology (Spanish: verb-framed, Swedish: satellite-framed, cf. Talmy, 2000), Swedish descriptions were substantially more consistent than Spanish descriptions. Swedish speakers almost invariably encoded all events with a single syntactic frame and systematically conveyed manner of motion. Spanish descriptions, in contrast, varied much more regarding syntactic framing and expression of manner. Crucially, variability in Spanish descriptions was not mainly a function of differences between events, as predicted by the event-properties account. Rather, Spanish variability in syntactic framing was driven by speaker biases. A similar picture arose for whether Spanish descriptions expressed manner information or not: Even after accounting for the effect of syntactic choice, a large portion of the variance in Spanish manner encoding remained attributable to differences among speakers. The results show that consistency in motion event encoding starkly differs across languages: Some languages (like Swedish) bias their speakers toward a particular linguistic event schema much more than others (like Spanish). Implications of these findings are discussed with respect to the typology of event framing, theories on the relationship between language and thought, and speech planning. In addition, the tools employed here to quantify variability can be applied to other domains of language.

## 1. Introduction

To what extent does our particular language constrain how we describe and conceptualize complex events? While a key property of language is that an unlimited number of ideas can be generated with finite means (Chomsky, 2002), a growing literature shows that particular languages also impose biases on *what* speakers express and *how* they express it. Cross-linguistic differences of this kind have enjoyed substantial theoretical interest in recent decades, largely as a consequence of cognitive linguistic approaches to language and their central tenet that “grammar reduces to the structuring and symbolization of conceptual content” (Langacker, 1999, p. 1) as well as their emphasis on subtle aspects of how we conceive of events (Goldberg, 2003). In this view, cross-linguistic differences may tell us something fundamental about how the world is conceptualized as a function of language (e.g., Lucy, 1992; Slobin, 1996; Wolff and Holmes, 2011; Boroditsky, 2012). Previous cross-linguistic descriptions of event encoding have mostly sought generalizations at the level of whole languages. In contrast, the aim of the present work is to focus on variability of event encoding patterns within languages, in line with the idea that variability in language is not simply noise but instead contributes valuable information for theoretical development (e.g., Dabrowska, 2012, 2016; Verhagen and Mos, 2016; Cunnings and Fujita, 2020; Verhagen et al., 2020).

The cross-linguistic contrast studied here concerns lexicalization patterns in motion event encoding, i.e., differences in which conceptual information of an event is linguistically expressed and how it is packaged into syntactic structure (Talmy, 1991, 2000; Berman and Slobin, 1994; for review, see Filipović and Ibarretxe-Antuñano, 2015). Languages can be classified into types depending on how they characteristically encode the fundamental motion component of path, i.e., the trajectory followed by the moving entity with respect to a landmark (Talmy, 2000). In satellite-framed (henceforth S) languages like Swedish, path is expressed outside of the main verb root.^1^ In contrast, verb-framed (henceforth V) languages like Spanish characteristically encode path in the main verb root (Talmy, 2000).

**Figure 1:**
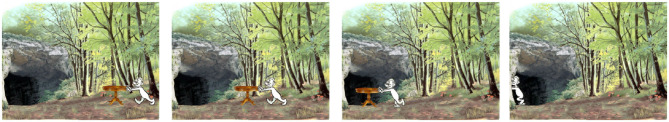
Sequence of stills taken from a stimulus event in the present study.

To illustrate, example (1) gives a typical Swedish S-description of the event shown in Figure 1. Path is expressed outside of the main verb root, in the prepositional phrase *in i grottan* “into the cave.” The main verb *skjuter* (“pushes”) conveys information about manner, i.e., the particular way in which motion is brought about or carried out. Since main verbs are syntactically obligatory, S-languages exhibit a strong tendency to express manner in motion descriptions (Slobin and Hoiting, 1994; Slobin, 2004; Slobin et al., 2014). In contrast, Spanish descriptions of the same event tend to be verb-framed (V): Path is expressed in the main verb root, as in (2) (*entra* “moves into”/“enters”). Because the main verb slot is taken up by the path verb, manner—if at all mentioned—needs to be encoded elsewhere. One option is to express manner in an adjunct, as in the gerund form *empujando* (“pushing”), in (2). However, manner is also frequently omitted in Spanish and other V-languages, as in (3), where the preposition *con* (“with”) leaves manner unspecified.


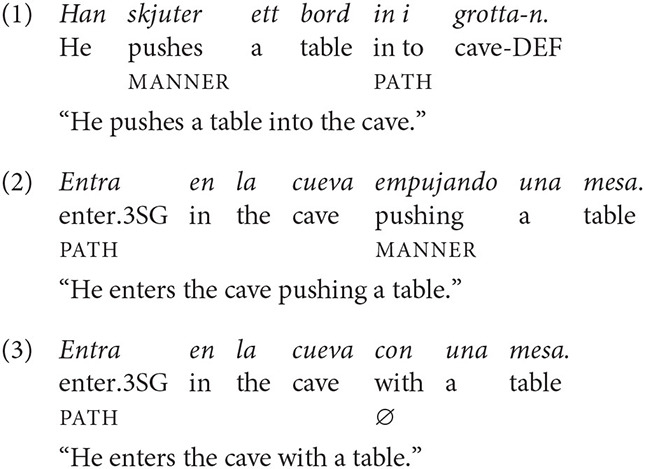


The previous distinction refers to “typical” or “characteristic” encoding patterns in a language. However, it is now widely recognized that a strict typology is somewhat of a theoretical straitjacket because of considerable within-language variability (Matsumoto, 2003; Slobin, 2004; Kopecka, 2006; Bohnemeyer et al., 2007; Filipović, 2007; Nikitina, 2008; Ibarretxe-Antuñano, 2009; Beavers et al., 2010; Croft et al., 2010; Slobin et al., 2011; Goschler and Stefanowitsch, 2013; Verkerk, 2014).^2^ English, for instance, is considered an S-language but it has a set of Latinate verbs that denote path of motion, such as *cross* or *ascend*, thus allowing for V-constructions (e.g., “he *enters* the cave”). Similarly, even though Italian is predominantly a V-language, it has a system of satellite-like verb particles like *giu* “down” or *via* “away” that encode path and combine with manner verbs very much as in canonical S-languages (Iacobini and Masini, 2006). What explains this variability?

Here we test a prominent account in the literature of why there is within-language variability, which we call the *event-properties* account. It is most explicitly formulated in Croft et al. (2010) and states that variability can be explained by specific properties of the events, such as whether an action reaches a goal or whether the type of motion is typical or atypical. The idea is that event framing patterns need not apply to a language as a whole but rather to complex event types (Croft et al., 2010). According to this account, some languages might apply a single pattern across the board (resulting in little overall variation), while others will fine-tune their syntactic patterns to particular events. In the latter case, what appears to be within-language variability is really just variability between event types (Croft et al., 2010).

The event-properties account seems compatible with previous observations in the literature. For example, Aske (1989) observed that constructions of the S-type are not impossible in Spanish in general, but only for events with telic paths, i.e., paths involving a change of location, such as crossing a physical boundary (e.g., entering a cave). However, the evidence for this “boundary-crossing constraint” (Slobin and Hoiting, 1994; Slobin, 2004) in V-languages is mixed (Naigles et al., 1998; Kopecka, 2009; Iacobini and Vergaro, 2014; Martínez Vázquez, 2015). Relatedly, Slobin has argued that manner encoding in V-languages depends on the salience of the manner component in the event, such that manner is specified only when the pattern of movement is really at issue (Slobin, 2005). In the same vein, Papafragou et al. (2006) found that whether or not Greek speakers encoded manner in their event descriptions depended on how inferable manner was, omitting it only when manner was obvious from the context (e.g., a man *walking* up the stairs), but not when it was not (e.g., a man *crawling* up the stairs).

**Figure 2:**
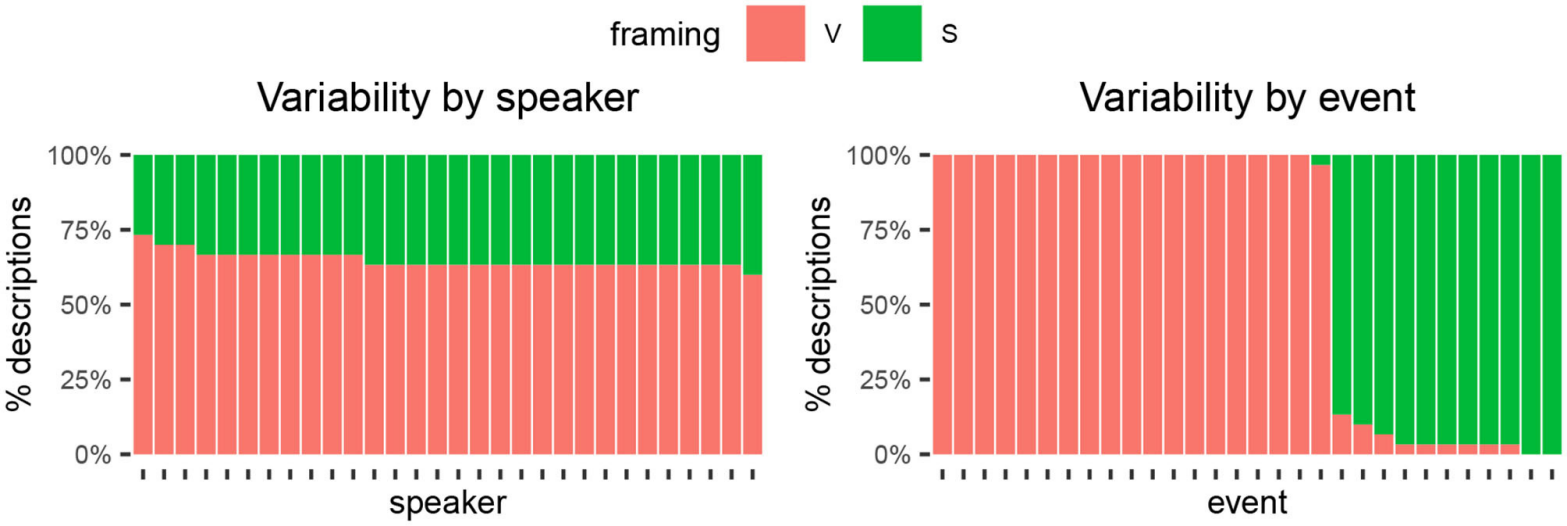
Hypothetical scenario in line with the event-properties account, where within-language variability is mostly explained by event properties (speakers play a minor role). (Left) Each column represents a speaker. (Right) Each column represents an event. Fill color shows percentage of descriptions following each framing pattern, either by speaker (left) or by event (right). In this scenario, consistency between speakers is high when describing the same event.

**Figure 3:**
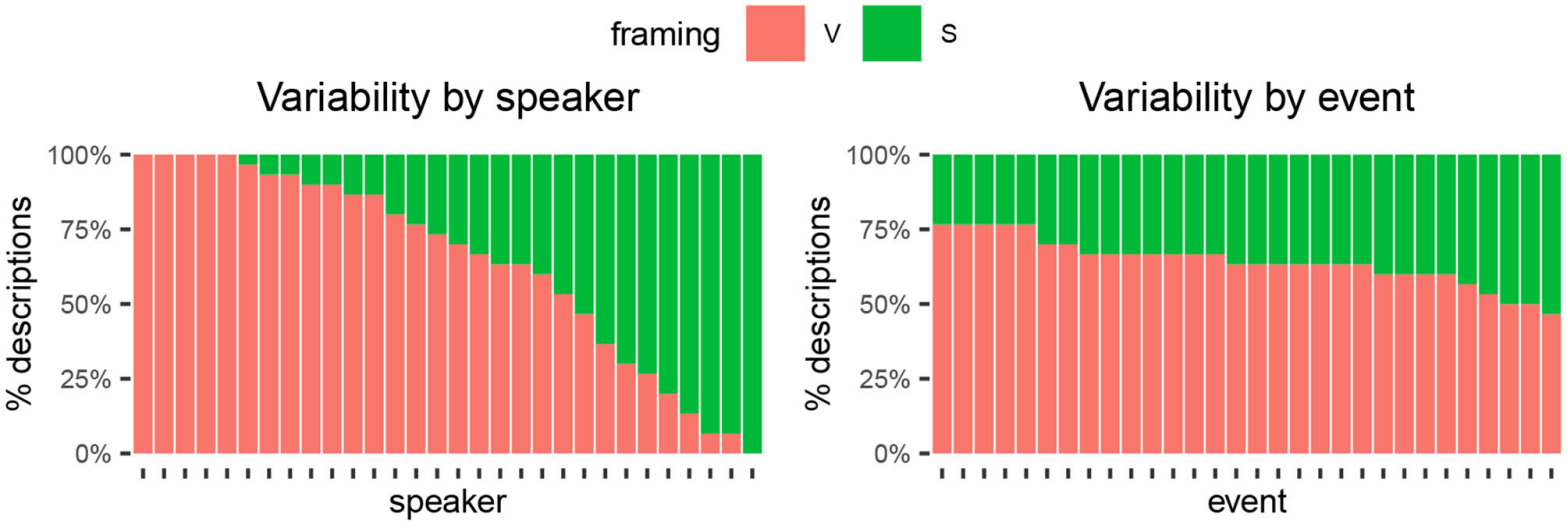
Hypothetical scenario against the event-properties account: Variability is mostly due to differences between speakers (event properties play a minor role). In this scenario, consistency between speakers is low when describing the same event. For figure interpretation, see caption in Figure 2.

Under the event-properties account, speakers of the same language have consistent ways of describing an event, even if differences arise between event types. An alternative to this is that some languages lack a systematic way of encoding events. Both of these alternatives can result in within-language variability and only an analysis of the sources of variability can adjudicate between the two. To illustrate, Figures 2, 3 depict two hypothetical languages, in both of which the proportion of V- and S-descriptions is identical (70 and 30%, respectively). Figure 2 would support the event-properties account: some events predominantly elicit V-descriptions while others strongly elicit S-descriptions (right panel). Variability by speaker would be relatively minor, with most speakers clustering around the language average (left panel). Such a scenario would indicate that speakers systematically activate event-specific linguistic templates (cf., Goldberg, 1995). In contrast, Figure 3 shows a scenario in which some speakers show a strong V-preference, others show an S-preference, and the rest falls somewhere in between (Figure 3, left panel). The events may still bias descriptions toward V or S to some extent, but their role is relatively minor: Any particular event elicits some V- and some S-descriptions, with proportions close to the language average (Figure 3, right panel). Against the event-properties account, this scenario would suggest that no particular pattern of event framing is strongly associated with motion events, not even when controlling for individual events.^3^

Teasing apart these two scenarios has important consequences for theories on the relation between language and thought and, in particular, for linguistic relativity, a topic that has sparked considerable interest in the last decades (Boroditsky, 2012; Casasanto, 2016; Bylund, 2019). Linguistic relativity posits a relation between an individual's language and their conceptual representation of the world (Whorf, 1956; Lucy, 1992; Levinson, 2003). The two necessary assumptions are that languages systematically differ in how they linguistically categorize the world and that language affects thought. From these two assumptions, it follows that speakers of different languages will think differently (Swoyer, 2011). However, if there is little consistency between how speakers describe the same events (as in Figure 3), this clearly undermines the first assumption that languages provide a systematic categorization of the world, and thus weakens the case for relativistic effects (cf. Kay, 1996).

Indeed, variability between speaker descriptions gives a measure of how codable a concept or event is in a language (e.g., Brown and Lenneberg, 1954; Majid et al., 2018). Highly codable events are consistently described within a language community (i.e., there is low between-speaker variability). One of the first experimental tests of linguistic relativity was in fact centrally based on speaker variability: Brown and Lenneberg (1954) measured consistency in how speakers of the same language (English) named different patches of colors. They hypothesized that colors that were more consistently labeled (i.e., more codable) should lead to better performance on individuals' recognition memory, because accessible labels that were strongly associated to the stimulus would support memory processes. This hypothesis was corroborated by their results (Brown and Lenneberg, 1954).

Surprisingly, although Talmy's typology has been applied to predict cognitive phenomena, little attention has been paid to speaker variability.^4^ The general approach of dividing languages into types, like S- and V-languages (and other types), may be appropriate from a purely descriptive point of view that only attends to linguistic structural patterns, which was arguably the main motivation in Talmy (2000). However, if we wish to predict general cognitive patterns that might correlate with certain linguistic habits, as in linguistic relativity research, it is clearly of relevance to know how tight the link is between the situation to be described and the linguistic schema by which it is described.

## 2. The Present Study

The aim of the present study is to examine variability in motion encoding across and within languages as a window onto linguistic event representation. We test the event-properties account, according to which variability should be a function of event properties and thus there should be only little within-language variability for each event.

We elicited caused motion descriptions of the same events and under identical experimental conditions from comparable populations of Spanish and Swedish speakers. Spanish and Swedish are generally considered prototypical cases of a V- and an S-language, respectively, and thus provide a useful comparison. The choice of caused motion is motivated by previous studies that have qualitatively suggested that there is variability in how this type of event is described in French (a V-language), but not in English (an S-language) (Hendriks et al., 2008; Hickmann and Hendriks, 2010). Since variability itself was the phenomenon of interest, speaker sample sizes were larger than in most previous studies (42 speakers per language, more than doubling typical samples of 12–20 speakers).

We first verify that the two languages indeed show the typologically expected pattern at the aggregated level of language: a V-preference in Spanish and an S-preference in Swedish. Next, we examine in closer detail variability in event framing to test how well event properties can account for it. Lastly, we turn to variability in the expression of manner information and assess how well it fits with the event-properties account.

To gain a detailed understanding of the sources of variability in the data, we use visualizations, the information-theoretical notion of entropy, and Bayesian mixed models. To allow for similar analyses in future work, all data and R-based scripts necessary for reproducing the results are publicly shared through a Dataverse repository (see Data Availability Statement).

## 3. Methods

### 3.1. Participants

The participants were 42 native Spanish speakers of the Peninsular variety (*M*_age_ = 23.7, *SD* = 3.2; 25 females) and 42 native Swedish speakers (*M*_age_ = 23.9, *SD* = 3.8; 25 females). Spanish and Swedish participants were recruited among university students at the Universidad Complutense Madrid (Spain) and at Stockholm University (Sweden), respectively. All participants used their first language routinely. None of them reported being bilingual from birth nor having any expert knowledge in a foreign language.^5^

### 3.2. Stimuli

The target events consisted of 32 video animations, each ~7 s long, originally designed by Hickmann and Hendriks (2010). In each event, the same agent moved different objects in different manners and along different paths. Several aspects of the events were systematically crossed in the stimuli, namely the manner in which the agent caused the object to move (pushing or pulling), the way in which the object itself moved (rolling or sliding) and the path followed by agent and object (up, down, into or across a landmark). For each combination of these values, there were two events: in one of them motion proceeded from left to right, and in the other, from right to left. The events comprised eight different landmarks (two per path) and 16 different objects (four per manner of cause and manner of object combination). See the Data Availability Statement for a full description of the target events. There were also seven distractor items that showed unrelated motion events in which inanimate objects moved along different trajectories, and one training item similar to the target events.

### 3.3. Procedure

All participants were tested individually in a quiet room after providing informed consent. They were told that they would see a figure called Hopi (“Popi” in Spanish, “Hoppi” in Swedish) doing different things and that their task was to describe what had happened in each scene after the animation had played in its entirety.^6^ Great care was taken to not prime the participants with any example sentences. No strict limitations were given as to the length of the descriptions, but participants were told to focus on “what happened” rather than on the details of the scenery. All participants started describing the training item, which served to introduce the agent and clarify any questions they might have. The target events were played in four semi-randomized lists counterbalanced across participants, with the seven distractor items interspersed at regular intervals. Descriptions were audio-recorded for later transcription.

### 3.4. Coding

For each transcription, the target description comprised all clauses that referred to the dynamic motion event. The 2,688 target descriptions (1,344 per language) were coded for two dependent variables: framing strategy and manner encoding. Framing strategy was determined solely on the basis of where path was expressed (cf. Talmy, 2000), resulting in three possible values: (a) V-framed (V) if path was expressed in the main verb, (b) S-framed (S) if path was only expressed outside of the main verb, and (c) no path if path was not expressed. A path expression was any expression that conveyed one of the four path values in the stimuli: *up, down, into*, or *across*. Cases in which path was redundantly expressed in the main verb and elsewhere were treated as V (e.g., *sube para arriba* “he ascends up”). Targets that contained more than one main clause were treated as V if at least one of the main verbs expressed path (e.g., *empuja la mesa y la mete en la cueva* “he pushes the table and inserts [path] it in the cave”).

The second variable, manner encoding, was treated as a binary variable: a target description either expressed manner information or not. Manner was mostly conveyed as part of a verbal root (e.g., Swedish *puttar* “pushes”), but other means of conveying manner were also counted (e.g., Spanish *delante* “in front of him” in *sube con un regalo delante* “he ascends with a present in front of him”).

### 3.5. Entropy Computation

To quantify variability by speakers and items, we use entropy. The entropy *H* of a variable quantifies that variable's degree of randomness or variability (Cover and Thomas, 2005). High entropy values indicate high variability (high randomness), whereas entropy values close to zero indicate low variability (low randomness, i.e., a predictable outcome).

For a categorical variable like *Framing*, which can take on discrete values (S, V, or NoPath), the entropy is defined as

$$H\left(Framing\right)=-\underset{x\in X}{\sum }p\left(x\right)\log_{2}p\left(x\right),$$

where *x* denotes each of the three possible values of *Framing* and *p*(*x*) is its probability. In our analysis, we compute entropy by speakers and events as follows (see Data Availability Statement for example calculations).

For speakers, *p*(*x*) in the formula above is estimated with the proportion of events a participant described with a given framing. This entropy score per speaker is a measure of how variable that speaker's descriptions are. A speaker who follows a very consistent pattern (i.e., whose descriptions show little variability) will have entropy close to zero. In contrast, speakers who do not show consistent patterns will have high entropy. For each language, we obtain a distribution of speaker entropy scores.

We also compute entropy for each event in an analogous fashion: *p*(*x*) is now estimated with the proportion of speakers who described a given event using each framing. If the pattern with which an event is described is highly consistent, entropy will be close to zero; but if variability is high for that event, entropy will also be high. The prediction in the literature that within-language variability is explained by differences between event properties means that entropy values computed by events should be close to zero.

Entropy presents a number of desirable properties. First, it is a mathematically well-defined notion that enjoys growing use in the language sciences (e.g., Montemurro and Zanette, 2011; Gries, 2012). Second, entropy is flexible and can be computed for categorical variables with any number of levels as well as for continuous variables. Lastly, entropy is a direct quantification of variability that abstracts away from the actual patterns in the data. Thus, one can compare variability across languages even when their dominant patterns differ (e.g., S vs. V).

## 4. Results

### 4.1. Dominant Pattern of Event Framing per Language

Table 1 shows the proportion of descriptions adopting each framing strategy per language. As expected, the most common strategy was verb-framing (V) in Spanish and satellite-framing (S) in Swedish. However, these proportions already reveal a striking difference in consistency: Swedish descriptions were almost exclusively of the S-type (97%), whereas in Spanish only 59% of descriptions followed the language-dominant V-pattern.

**Table 1:** Framing strategies in each language.

**Framing**	**Definition**	**Spanish %**	**Swedish %**
V	Path in main verb root	59	0
S	Path outside of main verb root only	35	97
No Path	Path not expressed or underspecified	6	2

The template of a typical Swedish description is given in (4): a transitive construction in which the main verb expressed manner (push, roll, etc.) and path was encoded in the prepositions of a directional prepositional phrase (into, over, etc.). An example is shown in (5). A related S-pattern involved the use of path particles immediately after the verb followed by the object NP and then a prepositional phrase, as in (6).


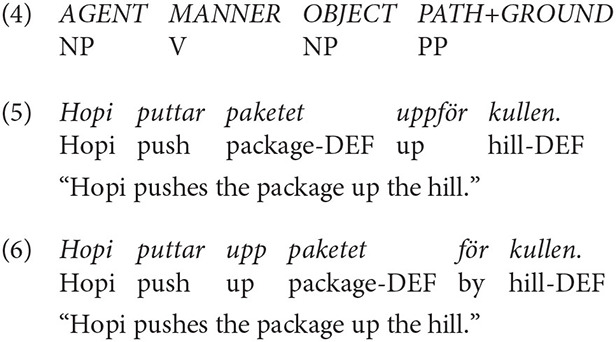


In Spanish, the two patterns (V and S) were frequent across descriptions (59% and 35%, respectively), even though V-framing dominated. An example of a Spanish V-description is shown in (7) and one of a S-description, encoding manner in the main verb and path in a prepositional phrase, is given in (8).


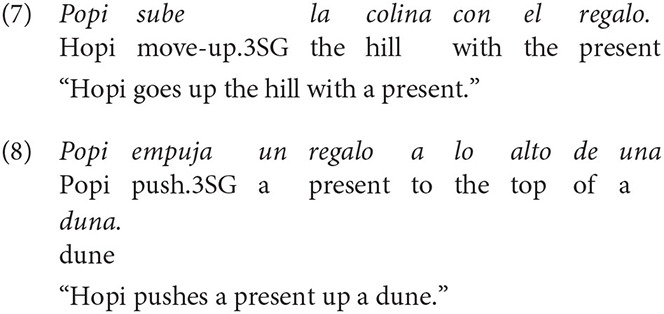


### 4.2. Variability by Speakers and Items

**Figure 4:**
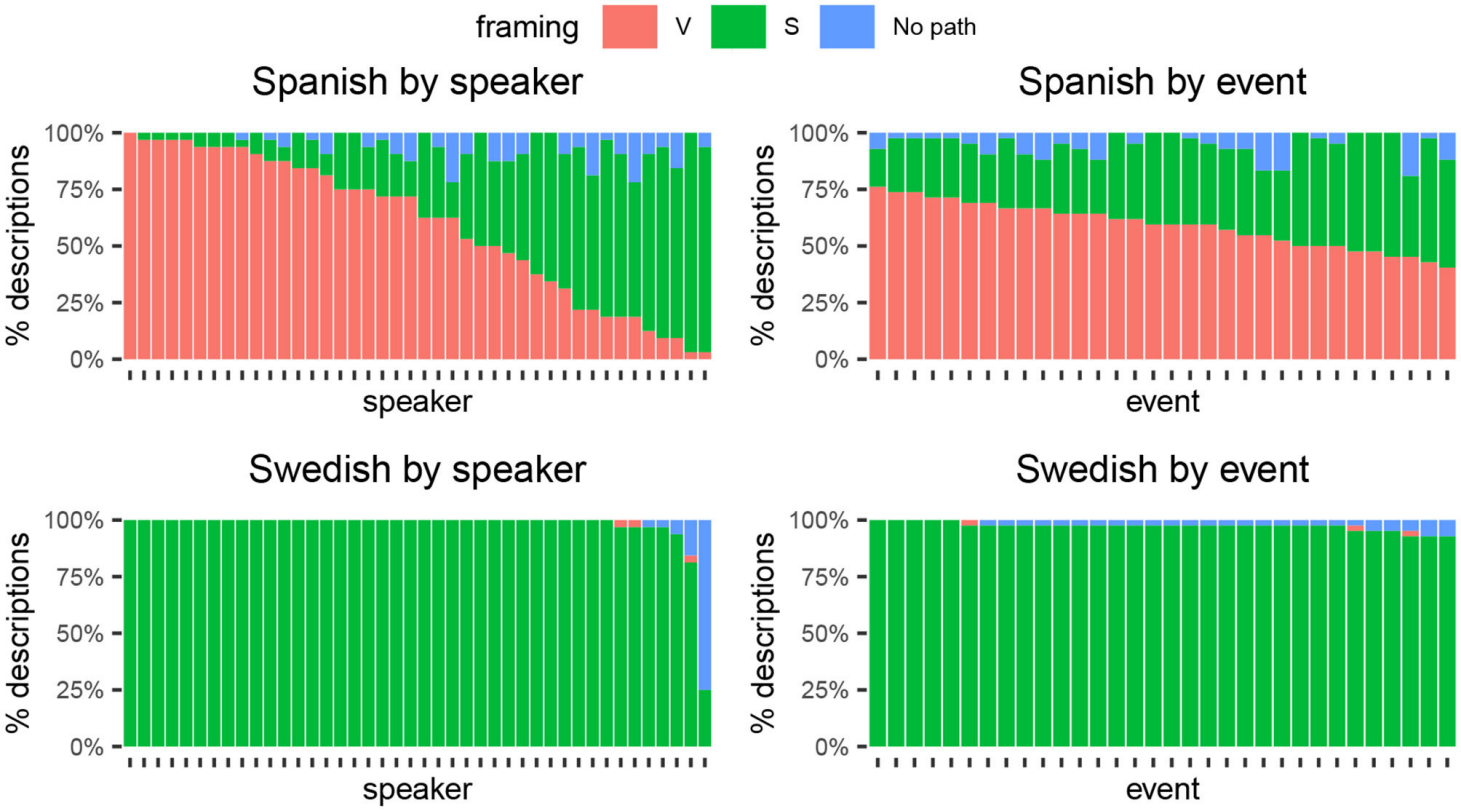
Framing strategies broken down by speakers and events (left vs. right) in Spanish and Swedish (top vs. bottom). Columns in the left panels represent speakers and their fill color shows the percentage of descriptions following a given framing strategy. Columns in the right panels represent events and their fill shows the percentage of participants who described that event with each framing strategy.

Figure 4 reveals the source of variability in each language by breaking down framing strategies by speakers and events. Top panels show the Spanish, and lower panels show the Swedish data. The left panels break down the data by speakers: Each column along the x-axis represents a speaker and its fill shows the proportion of descriptions that follow each event framing. Analogously, the right panels show variability by events, with columns representing events and fills showing the proportion of speakers who described each event with each framing strategy.

For Spanish, Figure 4 (top panels) suggests that variability between speakers was substantially larger than variability between events. The top left panel shows that some Spanish speakers consistently used V-framing as in example (7) (columns at the left end), while others almost exclusively used S-framing as in (8) (columns at the right end). The rest of the speakers fell somewhere along this spectrum. In contrast, the top right panel shows that differences between events in Spanish descriptions were less marked: No event exclusively elicited one type of framing strategy or another. Instead, each event to some extent was described with V- or S-framing.

The low consistency in how individual events were framed speaks against the event-properties account and Croft et al.'s (2010) prediction that Talmy's typology would apply to “individual complex event types within a language” (p. 202). The data show that even for one and the same event there can be substantial variability in how different speakers of the same language syntactically frame it, that is, no consistent pattern arises in Spanish, even when looking at events individually.

For Swedish, in contrast, the lower panels in Figure 4 show that variability was low both by speakers and by events, which follows from the fact that the pattern was highly consistent at the group level (Table 1). Descriptions followed the S-patterns exemplified in (5) and (6). Interestingly, the little variability that is found in Swedish descriptions is introduced by a few occasionally divergent speakers, rather than by some oddly described events, against the predictions of the event-properties account.^7^

### 4.3. Quantifying Variability: Entropy Analysis

Entropy is a suitable measure to *quantify* variability—or conversely, consistency—by speakers and events (see section 3.5 for methodological details). The prevalent idea in the literature that differences between events explain within-language variability implies that there should be high consistency in how events are described within a language, i.e., that entropy by events should be low.

**Figure 5:**
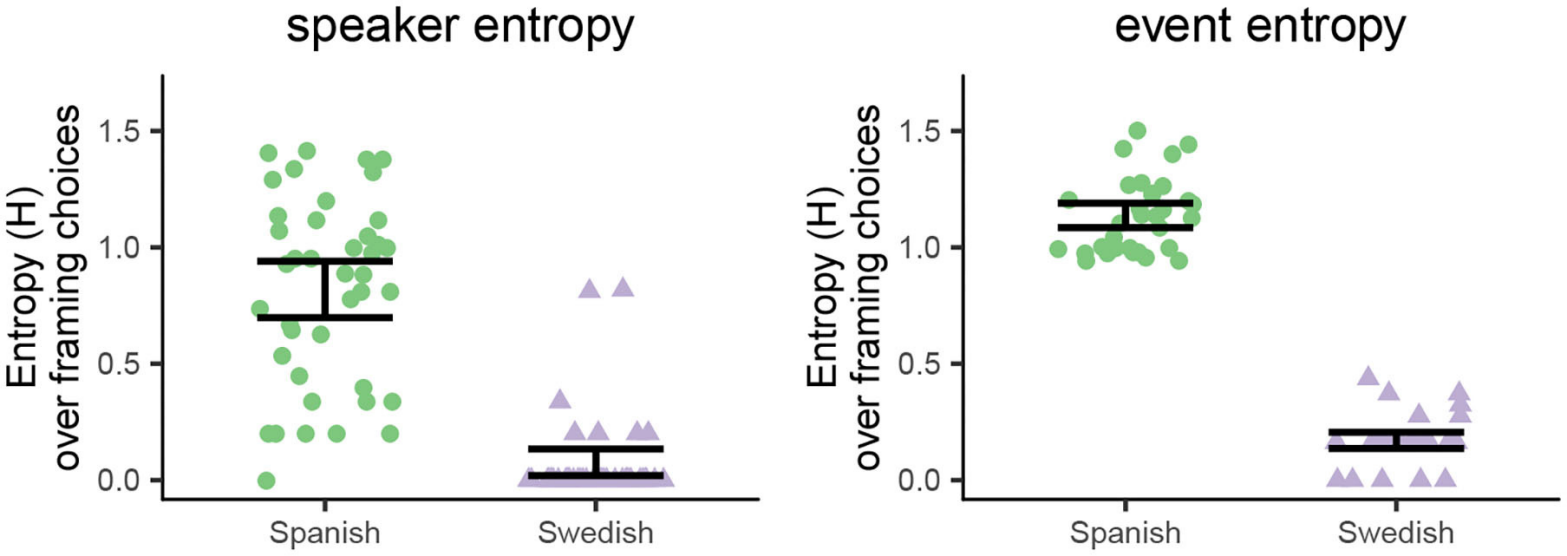
Entropy over framing strategies in each language, computed by speakers (left) and events (right). High entropy indicates high variability, and low entropy indicates low variability. Green dots show Spanish data and purple triangles Swedish data. Entropy scores are jittered along the x-axis and error bars show non-parametric 95% confidence intervals.

Figure 5 shows entropy over event framing in both languages, computed by speakers and events (left and right panels, respectively). Swedish speakers mostly had zero or near-zero entropy values (left panel—purple triangles), which reflects their consistent use of the same S-pattern. Spanish speakers, on the other hand, showed a much wider distribution of entropy scores (left panel—green dots): Speakers with low entropy values correspond to those who were consistent in their framing choices, sticking to either V- or S-framing. Speakers with the highest entropy values are those who were about equally likely to use any of the three framing patterns (V, S, or no path) and were thus most unpredictable. A Mann–Whitney test indicated that entropy by speaker was reliably larger in Spanish (*Mdn* = 0.9) than in Swedish (*Mdn* = 0), *W* = 1, 688, *p* < 0.001.^8^

The interpretation of entropy by events is analogous (Figure 5, right panel): Entropy over event framing was consistently low in Swedish, as there was little variability in how each event was described. This contrasts with the high entropy values by event in Spanish descriptions, showing that all events were described with variable patterns. A Wilcoxon matched-pairs signed-rank test indicated that entropy over event framing was reliably greater in Spanish (*Mdn* = 1.1) than in Swedish (*Mdn* = 0.2), *V* = 528, *p* < 0.001.

In sum, variability was greater in Spanish than in Swedish, both by speakers and events: The Spanish data show high entropy by events in combination with a wide spread of entropy by speakers. This means that the overall language-level variability observed in Spanish does not simply depend on event properties, because otherwise entropy by events should be low. Instead, it is largely due to differences between speakers, showing that no single framing strategy is consistently used by Spanish speakers.

### 4.4. Variability in Manner Encoding

So far we have focused on framing strategy, that is, the syntactic packaging of the path component. We now turn to the other aspect of motion descriptions that differs between S- and V-languages, namely the encoding of manner. In a similar fashion as before, we examine patterns of variability in whether or not manner information is expressed. If it is the case that manner encoding in V-languages depends on the salience (Slobin, 2005) or inferability (Papafragou et al., 2006) of the manner component in the event, then variability by events should be low in Spanish: For any given event, manner should be specified if it is salient or non-inferable—and otherwise omitted.

**Figure 6:**
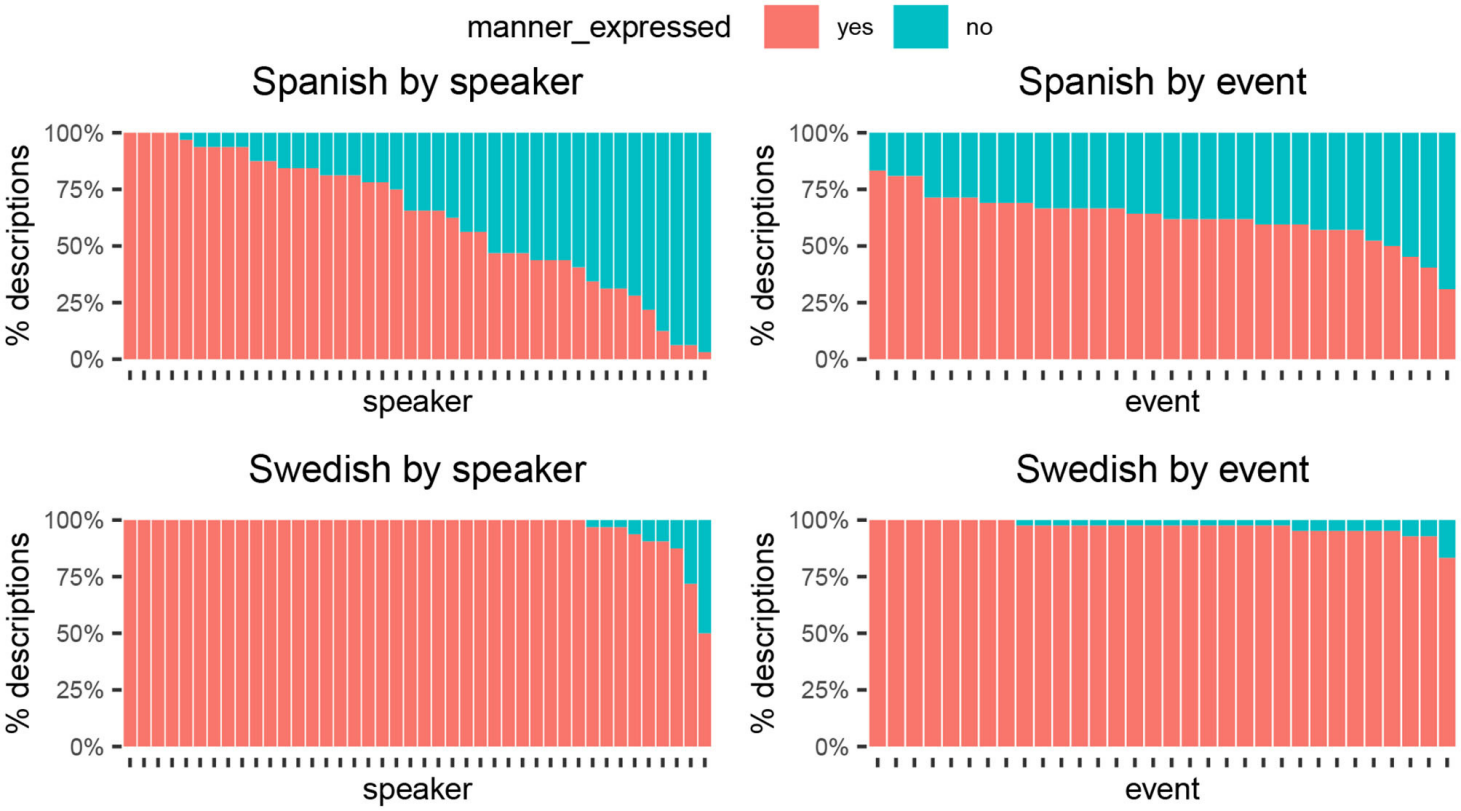
Manner encoding broken down by speakers and events (left vs. right) in Spanish and Swedish (top vs. bottom). For interpretation, see caption in Figure 4 and main text.

**Figure 7:**
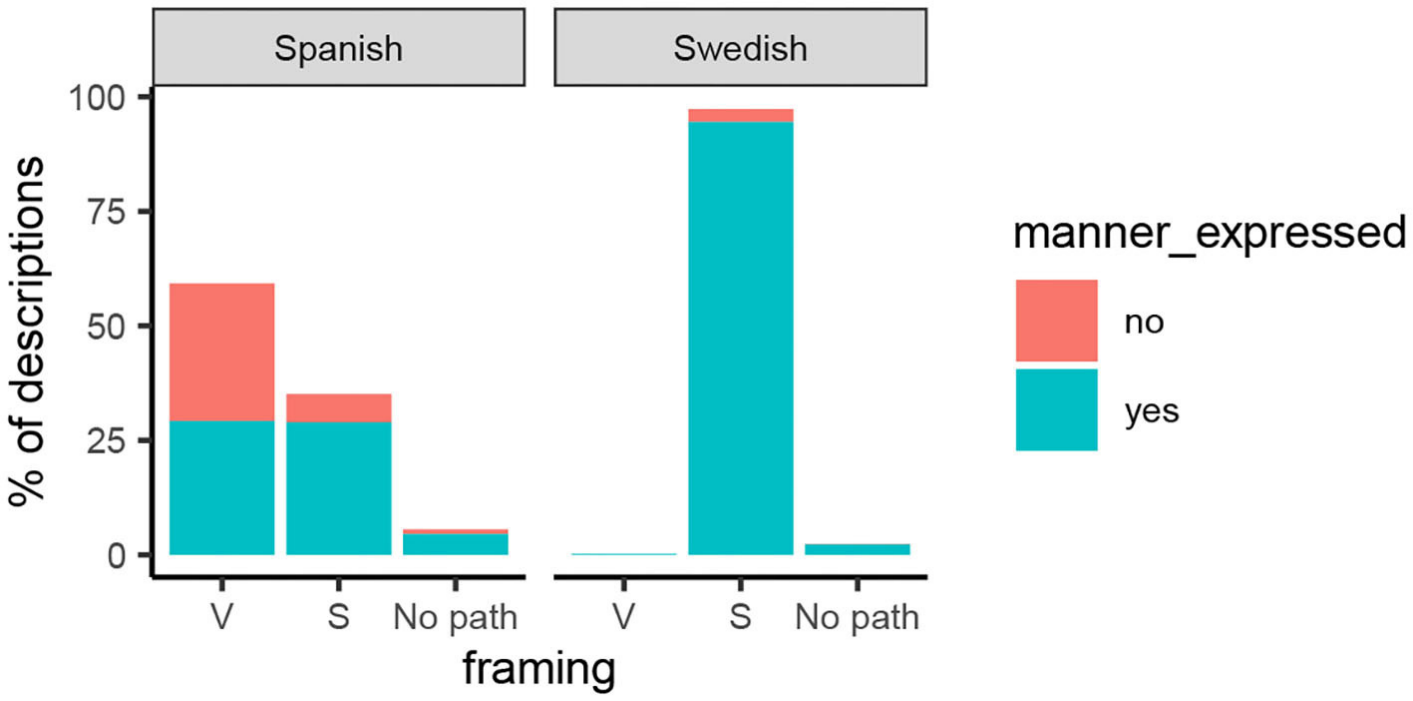
Percentage of descriptions in each language that expressed manner (fill color) as a function of framing strategy.

Overall, Swedish speakers mentioned manner very consistently: 97% of descriptions did so, as in examples (1), (5), and (6). The pattern was much more mixed in Spanish: 63% of descriptions did mention manner, as in examples (2) and (8), while 37% did not, as in (3) or (7). Figure 6 shows speaker and event variability in manner encoding in Spanish (top panels) and Swedish (bottom panels). The picture is strikingly similar to what we found for event framing. In Spanish, speakers were the largest source of variability: Some Spanish speakers had a strong preference to encode manner, others an almost equally strong preference to omit it, and most fell somewhere in between (top left panel). Differences between events were less pronounced and mostly clustered tightly about the language mean (top right panel). Swedish speakers, on the other hand, consistently encoded manner (Figure 6, bottom left panel), which follows from the overall language proportions (see Figure 7). Note that the little variability present in the Swedish data was also introduced by speakers rather than by events, as was the case for event framing.

In sum, because by design manner is held constant within each event, the high variability by events in Spanish runs counter to the event-properties account. Again, the data show that Spanish speakers, in contrast to Swedish speakers, do not consistently choose to encode the same *semantic content* (specifically, manner) for one and the same event.

### 4.5. Expression of Manner as a Function of Framing Strategy

To understand how manner encoding relates to framing strategy, Figure 7 plots the percentage of descriptions expressing manner in each language as a function of framing. For Swedish (right panel), it shows, as expected, that there is little variability with respect to both framing strategy (typically S) and manner encoding (typically expressed). For Spanish (left panel), on the other hand, it suggests that framing strategy was a predictor of manner encoding: Approximately half (49%) of Spanish V-descriptions contained manner information, as in example (2), whereas the other half did not [see example (3)]. In contrast, most of the Spanish S-descriptions (82%) did convey manner, as in example (8). Across languages, descriptions that did not express path (“no path”) were rare (4%); most of them (85%) tended to include manner information (see text footnote 7 for an example).

To confirm the relation between framing strategy and manner encoding in Spanish (Figure 7, left panel), we fitted a Bayesian logistic mixed model on the Spanish data, using the R package *brms* v. 2.10.0 (Bükner, 2017). Manner was the binary dependent variable (1= expressed, 0= not expressed) and framing strategy was the only fixed effects predictor (contrast coded: −1= V, 1= S); “no path” descriptions were excluded from this analysis (5.6%). The random effects included by-speaker intercepts and slopes for framing, and by-event intercepts and slopes for framing (i.e., it was the maximal model defined by the design, see Barr et al., 2013). We used *brm*'s default, non-informative priors for fixed and random effects. For details of model fitting, see Data Availability Statement.

This analysis indicated that framing strategy indeed was a significant predictor of manner encoding in Spanish: log-odds of expressing manner for S- vs. V-framing = 1.51, *SE* = 0.27 (95% Bayesian credible interval = [1.00, 2.09]).^9^ Thus, on average, S-framing made manner encoding 4.5 times more likely than V-framing in Spanish.

The Bayesian logistic mixed model additionally allows us to examine whether speakers or events were associated with larger variability on manner encoding, after accounting for the population-level effect of syntactic framing. This information is captured in the model's variance parameters associated to each of the random effects (Schielzeth and Nakagawa, 2013). Variance associated with speakers was consistently larger than that associated with events, both regarding random intercepts (by-speaker intercepts: standard deviation [SD] = 2.37, *SE* = 0.39 vs. by-event intercepts: SD = 0.78, *SE* = 0.16) and random slopes for framing (by-speaker slopes: SD = 1.10, *SE* = 0.27 vs. by-event slopes: SD = 0.29, *SE* = 0.15). Thus, the SD associated with speakers was more than three times larger than that associated with events.

In sum, framing strategy was a statistical predictor of manner encoding in Spanish. Once this effect was accounted for, however, speaker variance still accounted for much more of the variability in the data than variance associated with the events, further supporting the lack of a consistent pattern to describe motion events in Spanish.

## 5. Discussion

The present study on cross-linguistic representation of motion events focused on variability in event encoding. We evaluated the event-properties account of within-language variability, which states that “Talmy's typological classification applies to individual complex event types within a language, not to languages as a whole” (Croft et al., 2010, p. 202). We tested the prediction that follows from this account, namely that descriptions of the same event should be consistent within a language. Each of the two languages we compared is generally taken to represent one dominant type of motion encoding: Spanish, a V-language (Talmy, 2000), and Swedish, an S-language (Ragnarsdóttir and Strömqvist, 2004; Gullberg and Burenhult, 2012).

First, we found that already at the group level Spanish and Swedish showed a striking difference in variability: Swedish descriptions were almost altogether consistent in following the S-pattern and systematically conveying manner information, but Spanish descriptions varied greatly with regard to framing strategy and manner encoding, replicating similar earlier results in French (Hendriks et al., 2008).

Critically, we found little evidence for the event-properties account of within-language variability. Instead, the data show that variability arises because no single framing pattern is consistently applied among Spanish speakers, even for one and the same event. That is, most of the variability in Spanish was not the result of some events consistently eliciting V-descriptions and others S-descriptions, or some events eliciting manner in the descriptions and others not. Instead, it was the speakers who varied substantially. Spanish speakers formed a spectrum in their individual preferences for framing strategy, ranging from those who only produced V-descriptions to those who almost exclusively used the S-pattern; most participants used both patterns to some extent, but the choice of one or the other was not systematically linked to specific events. A similar gradient of preferences was found in manner encoding, from speakers who always expressed manner to those who hardly ever did. Even after controlling for the effect of framing strategy on manner encoding in a logistic mixed model, speakers emerged as a substantially larger source of variability than events.

### 5.1. Typologies of Motion: How Entrenched Are Framing Patterns?

The results show that Swedish is much more accurately characterized as an S-language than Spanish is as a V-language when it comes to the description of caused motion. Classifying languages into a typology still captures general trends: the majority of Spanish descriptions were indeed verb-framed. However, the lack of a consistent syntactic framing in Spanish, even for one and the same event, demonstrates that for Spanish speakers there is no strong association between motion events and a linguistic schema to encode them.

In this sense, speaker variability offers an index of how entrenched an abstract linguistic pattern is among speakers of a language (cf. Dabrowska and Street, 2006), where entrenchment denotes the “degree to which the formation and activation of a cognitive unit is routinized and automated” (Schmid, 2007, p. 119). Highly entrenched constructions are automatically activated when certain situations in the world are to be described by a speaker (Langacker, 1999); they result in a “gestalt formation” (Divjak and Caldwell-Harris, 2015, p. 61) that links perceptual input and a target linguistic conceptualization.

Our data indicate that the S-pattern of describing motion events is deeply entrenched in Swedish—it is routinized and automated. When asked to describe a caused motion event, Swedish speakers retrieve a very specific linguistic construction (cf. Goldberg, 1995; Croft, 2001), which specifies the grammatical structure of the sentence and determines how semantic components like path and manner map onto the different syntactic slots. However, the same is not true for the V-pattern in Spanish. Spanish descriptions alternate in syntactic patterns and semantic content, not only between, but also within, speakers. In other words, no linguistic schema is deeply entrenched in Spanish.

The lack of attention to speaker variability in previous cross-linguistic work is surprising given that entrenchment is widely recognized as a psychological phenomenon that is essential to language (Langacker, 1999; Schmid, 2007, 2017; Ambridge et al., 2008; Caldwell-Harris et al., 2012; Divjak and Caldwell-Harris, 2015). As argued in Dabrowska (2016), ignoring individual variability comes at the cost of missing out on a “window onto the cognitive and experiential underpinnings of language” (p. 485). While classifying languages regarding their dominant lexicalization patterns has some descriptive value (Talmy, 2000), it is necessary to find out how consistently these patterns are applied, as this offers an index of the degree to which they are entrenched. In other words, typological descriptions need to quantify and characterize variability within a language. In what follows, we discuss two areas for which the current findings have implications and where a more refined quantification of variability will help theory development.

### 5.2. Implications for Linguistic Relativity

The current findings have several implications for the linguistic relativity hypothesis—the claim that the language we speak affects our mental categories (see Wolff and Holmes, 2011; Boroditsky, 2012; Gleitman and Papafragou, 2012). A mechanism commonly hypothesized to drive Whorfian effects is that language provides conceptual schemas that become automatized through repeated use, making them easily available also in situations where language is not used (Whorf, 1956; Lucy, 1992; Levinson, 2003). Assuming such a mechanism, a typological feature like the S/V-distinction is useful for testing relativistic hypotheses only to the extent it predicts the habitual linguistic experience of speakers of a language. But if speaker variability with respect to a feature is large *within* a language, as in the present case for Spanish, then power to detect the classical Whorfian effect *between* languages would be reduced even if it existed. The mixed findings in the literature on Whorfian effects in the motion domain (Gennari et al., 2002; Papafragou et al., 2002; Kersten et al., 2010; Papafragou and Selimis, 2010; Montero-Melis and Bylund, 2017) could be a consequence of this within-language variability (cf. Loucks and Pederson, 2011; Montero-Melis et al., 2017). Therefore, future research testing effects of linguistic relativity needs to not only find cross-linguistic contrasts where languages either follow pattern A or B, but also explicitly measure the consistency with which these patterns are applied in each language.

Large speaker variability also has an upside if tests of the Whorfian hypothesis instead embrace it. Rather than testing the Whorfian hypothesis between languages, differences might be sought at the level of speakers. The individual linguistic biases of a speaker—for example in terms of encoding manner—should then become the main predictor. This idea is not new (e.g., Brown and Lenneberg, 1954). It underlies all Whorfian research which employs training paradigms (e.g., Dolscheid et al., 2013) and much of the literature on relativistic effects in second language speakers, where linguistic proficiency is treated as the key predictor of performance on non-linguistic tasks (for an overview, see Bylund and Athanasopoulos, 2014). The approach of the present study is relevant to that literature, because it will allow researchers to spell out the conditions under which it would make sense to look at effects of language on cognition on a speaker-by-speaker level, rather than (or in addition to) at the level of language, namely when variability at the speaker level is large (cf. Cunnings and Fujita, 2020).

An interesting novel hypothesis based on the current results is that the degree of entrenchment itself could have effects on the mental representations speakers form of whole event categories. This is markedly different from the hypothesis tested in previous research on relativity effects in the motion domain (i.e., whether speakers of different languages pay more attention to path or manner). A highly entrenched construction for describing caused motion events, as the one found in Swedish, might result in a robust memory schema that defines a single abstract mental template for all these events (cf. Gilboa and Marlatte, 2017). This abstract family resemblance might have the consequence that Swedish speakers readily think of a set of events as belonging to the same kind, while this might not be obvious to speakers of Spanish, who lack a single entrenched linguistic construction to describe these events.

This predicted effect can be seen as an instance of “ontological Whorfianism,” whereby language invites us to group particulars into a single category that we would not group together were it not for language (Reines and Prinz, 2009). Because the neurobiology of schemas is increasingly well-understood (e.g., Gilboa and Marlatte, 2017; Heidlmayr et al., 2020), an exciting prospect is to test whether merely watching the same events elicits differential neural processing across speakers of different languages, indicative of a more schematic processing when an entrenched linguistic construction exists (as in Swedish) compared to when it does not (as in Spanish).

### 5.3. Variability as a Window Into Speech Planning

The large variability in Spanish descriptions implies that Spanish speakers are faced with substantial choices—both syntactic and semantic—when producing a description. This raises three questions about how this variability impacts speech planning.

First, does syntactic choice facilitate or inhibit speech planning for Spanish speakers? Two claims alternate in the literature: Some earlier evidence supports the notion that syntactic flexibility benefits speech planning, resulting in quicker speech latencies and fewer errors, in line with a flexible and incremental view of speech planning (Ferreira, 1996). However, there is also evidence to the contrary, i.e., that syntactic choice slows down production, in line with a competitive model of language production where having several options hinders production (Hwang and Kaiser, 2014). While the present study was not designed to contrast these two claims (e.g., we did not collect speech onset latencies), preliminary analyses of speech disfluencies reported elsewhere suggest that Spanish speakers overall made more speech errors than Swedish speakers (Montero-Melis et al., 2016). These analyses also showed that syntactic variability at the individual level was correlated with more speech errors, that is, those speakers who had higher entropy over syntactic frames (see section 4.3) were also more likely to produce speech errors, such as pauses or false starts (Montero-Melis et al., 2016). In sum, there is some preliminary evidence that Spanish speakers may pay the price of diminished fluency in speech production for having a more flexible way of describing caused motion.

Second, the variability in Spanish descriptions raises an interesting question regarding *thinking for speaking*, the idea that the particular grammatical choices favored by a language affect how we conceptualize an event while speaking (Slobin, 1996). Do Spanish speakers always *think* of manner but sometimes omit it in their descriptions—or do they omit manner (when they do so) *because* they did not even think of it? This question can be framed in terms of Levelt's (1989) model of speech production. According to this model, the first stage of speech planning happens in the “conceptualizer,” which establishes a pre-linguistic event model in which event structure and participants are defined but not yet mapped onto linguistic units. Only at the next stage, the “formulator” decides how to structure the sentence syntactically. The question, then, is whether variability in Spanish descriptions originates at the level of the conceptualizer (in line with, e.g., von Stutterheim and Nüse, 2003) or the formulator. Previous evidence from a non-linguistic task comparing Spanish and Swedish speakers indicates that Spanish speakers paid less attention to manner (Montero-Melis and Bylund, 2017), suggesting that the effect originates at the level of the conceptualizer. However, more direct tests of this hypothesis would be possible capitalizing on the between-speaker variability in Spanish. For example, current decoding approaches (see Haxby et al., 2014) would in principle allow us to gauge if brain activity prior to speech onset (i.e., during speech planning) encodes the specific manner of the event irrespective of whether manner is later expressed in speech or not. An affirmative answer would be evidence against an effect in the conceptualizer.

A final question is how the kind of variability seen in Spanish affects the creation of situation models, that is, “mental representations of the people, objects, locations, events, and actions described in a text” (Zwaan, 1999, p. 15).^10^ Accounts of how situation models are constructed differ regarding how much importance they assign to structural aspects of the linguistic message (see Zwaan, 2016). Some authors consider it a design feature of language that it underspecifies any situation that is described (Gleitman and Papafragou, 2012), while other accounts assume that the listener arrives at a different construal depending on subtle aspects of linguistic realization (Goldberg, 1995, 2003; Langacker, 1999). Thus, an interesting question is whether situation models are resistant to variability in the descriptions, both within and across languages. That is, do Spanish speakers build similar situation models when processing a motion description independently of the linguistic schemas they are generated from? And do the situation models of Spanish speakers differ in predictable ways from those of Swedish speakers? This question links language processing to linguistic relativity since if cross-linguistic differences lead to differences in conceptualizations, they do so arguably because different structural patterns give rise to different situation models.

### 5.4. The Relevance of (Quantifying) Variability

Recent years have seen a reappraisal of the importance of variability for linguistic theories, with increased emphasis on the role of experience and variation in forming linguistic representations (Bybee, 2010; Dabrowska, 2012; Ellis et al., 2013; Kapatsinski, 2014; Gries, 2015; Hoffmann et al., 2019; Verhagen et al., 2020). In this respect, work on event conceptualization has been lagging behind: Most studies have tended to focus on central tendencies in the form of language averages, and only exceptionally variability has been broken down by speakers (e.g., Berthele, 2013) or events (e.g., Cadierno et al., 2016). So while previous work may offer quite detailed *qualitative* reports of variability (e.g., Hickmann and Hendriks, 2010), a more thorough analysis of variability was needed.

The present study illustrated how to analyze variability and may prove useful beyond the domain of motion (see also Gries, 2012, 2015; Gries and Ellis, 2015). We used the information-theoretical notion of entropy and the variance components in (Bayesian) mixed models as two ways of quantifying the intuition of consistency across speakers and events. Entropy is a flexible and mathematically well-defined measure of variability, allowing for quantification and comparison of variability between languages. Here, we were able to show that Swedish is much more accurately characterized as an S-language than Spanish is as a V-language when it comes to the description of caused motion. More generally, our analysis approach could be a welcome addition in typology, as it will allow for a more flexible comparison of variability across languages. An advantage for use in cross-linguistic settings is that entropy can be computed over categorical variables with any number of levels, obviating the need to reduce different patterns to binary values. Thus, even in situations where a linguistic phenomenon is expressed in different ways across and within languages, entropy mathematically represents this variation as a single underlying distribution, whose variability or “randomness” can be straightforwardly quantified.

### 5.5. Limitations

As a qualificatory note, the present study comprised a limited set of caused motion events and focused only on two languages. To further generalize the current findings, it will be necessary to extend the present approach to other types of motion events and other languages. An open question is whether the lack of consistency reported here for Spanish holds more generally across V-languages. To address this, it will be necessary to test other V-languages and see if they pattern like Spanish. Also, the sampled participant populations were relatively homogeneous (in terms of age, regional and social background, level of education, etc.); more heterogeneous samples will be needed before definitive conclusions can be drawn about languages as a whole. Even more importantly, the current study measures variability in production only. Therefore, it is not possible to fully tease apart two scenarios that could give rise to the small variability observed in Swedish descriptions: Is it the language that strongly constrains the options of how these events can be syntactically framed because V-framing is not even possible? Or do speakers have different choices but still consistently choose S-framing? While it is clear that path verbs are rare in Swedish (and therefore the choices at least somewhat constrained), a proper evaluation of this issue requires collecting acceptability ratings for the different types of constructions as applied to each event.^11^

## 6. Conclusion

Variability in event encoding offers a window into the mental representation of event schemas. When it comes to within-language variability, the dominant view in the literature has been that event properties account for it, but this account had not been empirically tested in a controlled experimental design. The present study tested this claim and found little evidence supporting it. Swedish descriptions were found to be highly consistent, whereas Spanish ones were much more variable. The analyses show that Spanish descriptions are variable, not because they are fine-tuned to different events, but because there is no consistent way to describe the same events across speakers. This suggests that some languages have more entrenched linguistic structures than others to describe the very same events. A discrete typology obscures this relevant fact; therefore, the quantification of variability should be part and parcel of any typology at the language level. Entropy offers a suitable and flexible tool to quantify such variability and thus the analysis presented here can be adopted in future typological descriptions.

The present findings have implications for theories of event representation in language and cognition: Linguistic templates that are not deeply entrenched among speakers of a language are unlikely to result in strong mental schemas. A more fruitful approach to testing linguistic relativity effects in the domain of motion is thus to not focus exclusively on the dominant pattern of motion encoding, but rather on the fact that some languages have a highly entrenched pattern, while others do not. This may lead to differences in event ontology across speakers of different languages (cf. Reines and Prinz, 2009). Finally, we highlighted the implications of this lack of entrenchment for speech planning, suggesting that variability of the sort reported here can provide an effective test ground for open questions about the effects of syntactic choice on sentence planning. In sum, the present study contributes to the view (cf. Verhagen et al., 2020) that variability in language provides a valuable source of information that should be analyzed and interpreted rather than dismissed as noise.

## Data Availability Statement

The data sets and R scripts necessary to reproduce all analyses reported in this paper are accessible at https://doi.org/10.7910/DVN/Z12TY1.

The repository contains:

- README file explaining how to reproduce the analyses on your own computer.
- Basic data file in csv format.
- Item description in csv format.
- Supporting information: R-generated *knitr* report with supporting information and the code that generated all analyses reported in the paper (in html format).
- Files containing the R code necessary to generate the Supporting Information file.

## Ethics Statement

Ethical review and approval was not required for the study on human participants in accordance with the local legislation and institutional requirements. The patients/participants provided their written informed consent to participate in this study.

## Author Contributions

The author confirms being the sole contributor of this work and has approved it for publication.

## Conflict of Interest

The author declares that the research was conducted in the absence of any commercial or financial relationships that could be construed as a potential conflict of interest.

## Footnotes

^1^The present study follows several authors in adopting a broad notion of satellite as “anything that is not a verb root but encodes an event component” (Croft et al., 2010, p. 206; see Filipović, 2007; Beavers et al., 2010; Verkerk, 2014 for essentially the same operationalization).

^2^Other criticisms of a two-way typology include the fact that some languages follow an all-together different pattern (e.g., so-called equipollently framed languages, see Slobin, 2004; Zlatev and Yangklang, 2004), or the substantial variation observed among languages supposedly belonging to the same type (Hijazo-Gascón and Ibarretxe-Antuñano, 2013).

^3^The two scenarios above do not exhaust all possibilities. For example, some speakers could always employ V-constructions and others consistently use S-constructions, with very little variation in between. Such a bimodal distribution for speakers would indicate a systematic alternation, possibly related to dialectal or some other sociolinguistic factor.

^4^Individual differences have been looked at through the lens of multilingualism, for example as they correlate with second language (L2) proficiency (e.g., Brown and Gullberg, 2012) or in relation with dialectal variation in situations of language contact (Berthele, 2006, 2013). The current focus, however, is not on effects of multilingualism or sociolinguistic variables.

^5^As part of routine procedure of our lab, a measure of participants' English proficiency not reported here was collected through part one of the Oxford Quick Placement Test (Oxford University Press et al., 2001).

^6^Core instructions: “You will now see a series of short animations with a character called Hopi who does different things. Sometimes the animations do not show Hopi, but your task is always the same: You have to describe what happened in the scene after the animation has played in its entirety.”

^7^The rightmost column in the lower left panel in Figure 4 corresponds to a Swedish speaker who omitted path in most descriptions, as in *Hoppi skjuter ett bord framför sig* (“Hopi pushes a table in front of him”).

^8^Non-parametric tests are used because entropy is not normally distributed. The R code to generate these analyses can be found in the Data Availability Statement.

^9^In a Bayesian framework, a 95% credible interval means that the parameter of interest has a 95% probability of being contained in that interval (see, e.g., Kruschke and Liddell, 2018).

^10^We thank an anonymous reviewer for bringing up this idea.

^11^We thank an anonymous reviewer for this comment.

## Abbreviations

Linguistic: S: satellite-framed
V: verb-framed
NP: noun phrase
PP: prepositional phrase
Statistical: SD: standard deviation
SE: standard error.

## References

[B1] Ambridge B. Pine J. M. Rowland C. F. Young C. R. (2008). The effect of verb semantic class and verb frequency (entrenchment) on children's and adults' graded judgements of argument-structure overgeneralization errors. Cognition 106, 87–129. 10.1016/j.cognition.2006.12.015 17316595

[B2] Aske J. (1989). Path predicates in English and Spanish: a closer look, in Proceedings of the Berkeley Linguistic Society, Vol. 15 (Berkeley, CA), 1–14. 10.3765/bls.v15i0.1753

[B3] Barr D. J. Levy R. Scheepers C. Tily H. J. (2013). Random effects structure for confirmatory hypothesis testing: keep it maximal. J. Mem. Lang. 68, 255–278. 10.1016/j.jml.2012.11.001 24403724 PMC3881361

[B4] Beavers J. Levin B. Tham S. W. (2010). The typology of motion expressions revisited. J. Linguist. 46, 331–377. 10.1017/S0022226709990272

[B5] Berman R. A. Slobin D. I. (Eds.). (1994). Relating Events in Narrative: A Crosslinguistic Developmental Study. Hillsdale, NJ: Erlbaum.

[B6] Berthele R. (2006). Ort und Weg: die sprachliche Raumreferenz in Varietäten des Deutschen, Rätoromanischen und Französischen. Berlin: Walter de Gruyter.

[B7] Berthele R. (2013). Disentangling manner and path: evidence from varieties of German and Romance, in Variation and Change in the Encoding of Motion Events, eds Goschler J. Stefanowitsch A. (Amsterdam: John Benjamins), 55–75. 10.1075/hcp.41.03ber

[B8] Bohnemeyer J. Enfield N. J. Essegbey J. Ibarretxe-Antuñano I. Kita S. Lüpke F.. (2007). Principles of event segmentation in language: the case of motion events. Language 83, 495–532. 10.1353/lan.2007.0116

[B9] Boroditsky L. (2012). How the languages we speak shape the ways we think: the FAQs, in The Cambridge Handbook of Psycholinguistics, eds Spivey M. McRae K. Joanisse M. (Cambridge: Cambridge University Press), 615–632. 10.1017/CBO9781139029377.032

[B10] Brown A. Gullberg M. (2012). Multicompetence and native speaker variation in clausal packaging in Japanese. Sec. Lang. Res. 28, 415–442. 10.1177/0267658312455822

[B11] Brown R. W. Lenneberg E. H. (1954). A study in language and cognition. J. Abnorm. Soc. Psychol. 49, 454–462. 10.1037/h0057814 13174309

[B12] Bürkner P. C. (2017). brms: An R package for Bayesian multilevel models using Stan. J. Stat. Softw. 80, 1–28. 10.18637/jss.v080.i01

[B13] Bybee J. L. (2010). Language, Usage, and Cognition. Cambridge: Cambridge University Press.

[B14] Bylund E. (2019). Linguistic Relativity, in SAGE Research Methods Foundations, eds Atkinson P. Delamont S. Cernat A. Sakshaug J. W. Williams R. A.. 10.4135/9781526421036847208

[B15] Bylund E. Athanasopoulos P. (2014). Linguistic relativity in SLA: toward a new research program. Lang. Learn. 64, 952–985. 10.1111/lang.12080

[B16] Cadierno T. Ibarretxe-Antuñano I. Hijazo-Gascón A. (2016). Semantic categorization of placement verbs in L1 and L2 Danish and Spanish. Lang. Learn. 66, 191–223. 10.1111/lang.12153

[B17] Caldwell-Harris C. L. Berant J. Edelman S. (2012). Measuring mental entrenchment of phrases with perceptual identification, familiarity ratings, and corpus frequency statistics, in Frequency Effects in Language Representation, eds Divjak D. Gries S. (Berlin: De Gruyter Mouton), 165–194.

[B18] Casasanto D. (2016). Linguistic relativity, in The Routledge Handbook of Semantics, ed Riemer N. (New York, NY: Routledge), 158–174.

[B19] Chomsky N. (2002). On Nature and Language. Cambridge: Cambridge University Press.

[B20] Cover T. M. Thomas J. A. (2005). Elements of Information Theory. Hoboken, NJ: Wiley.

[B21] Croft W. (2001). Radical Construction Grammar: Syntactic Theory in Typological Perspective. New York, NY: Oxford University Press.

[B22] Croft W. Barddal J. Hollmann W. Sotirova V. Taoka C. (2010). Revising Talmy's typological classification of complex event constructions, in Contrastive Studies in Construction Grammar, ed Boas H. C. (Amsterdam: John Benjamins), 201–235. 10.1075/cal.10.09cro

[B23] Cunnings I. Fujita H. (2020). Quantifying individual differences in native and nonnative sentence processing. Appl. Psycholinguist. 1–21. 10.1017/S014271642000064832255882

[B24] Dabrowska E. (2012). Different speakers, different grammars: individual differences in native language attainment. Linguist. Approach. Bilingual. 2, 219–253. 10.1075/lab.2.3.01dab

[B25] Dabrowska E. (2016). Cognitive linguistics' seven deadly sins. Cogn. Linguist. 27, 479–491. 10.1515/cog-2016-0059

[B26] Dabrowska E. Street J. (2006). Individual differences in language attainment: comprehension of passive sentences by native and non-native English speakers. Lang. Sci. 28, 604–615. 10.1016/j.langsci.2005.11.014

[B27] Divjak D. Caldwell-Harris C. L. (2015). Frequency and entrenchment, in Handbook of Cognitive Linguistics, eds Dabrowska E. Divjak D. (Berlin: De Gruyter), 53–75. 10.1515/9783110292022-004

[B28] Dolscheid S. Shayan S. Majid A. Casasanto D. (2013). The thickness of musical pitch psychophysical evidence for linguistic relativity. Psychol. Sci. 24, 613–621. 10.1177/0956797612457374 23538914

[B29] Ellis N. C. O'Donnell M. B. Römer U. (2013). Usage-based language: investigating the latent structures that underpin acquisition. Lang. Learn. 63, 25–51. 10.1111/j.1467-9922.2012.00736.x

[B30] Ferreira V. S. (1996). Is it better to give than to donate? Syntactic flexibility in language production. J. Mem. Lang. 35, 724–755. 10.1006/jmla.1996.0038

[B31] Filipović L. (2007). Talking About Motion: A Crosslinguistic Investigation of Lexicalization Patterns. Amsterdam: John Benjamins.

[B32] Filipović L. Ibarretxe-Antuñano I. (2015). Motion, in Handbook of Cognitive Linguistics, eds Dabrowska E. Divjak D. (Berlin: De Gruyter), 527–546.

[B33] Gennari S. P. Sloman S. A. Malt B. C. Fitch W. T. (2002). Motion events in language and cognition. Cognition 83, 49–79. 10.1016/S0010-0277(01)00166-4 11814486

[B34] Gilboa A. Marlatte H. (2017). Neurobiology of schemas and schema-mediated memory. Trends Cogn. Sci. 21, 618–631. 10.1016/j.tics.2017.04.013 28551107

[B35] Gleitman L. Papafragou A. (2012). New perspectives on language and thought, in The Oxford Handbook of Thinking and Reasoning, eds Holyoak K. J. Morrison R. G. (Oxford: Oxford University Press), 543–568. 10.1093/oxfordhb/9780199734689.013.0028

[B36] Goldberg A. E. (1995). Constructions: A Construction Grammar Approach to Argument Structure. Cognitive Theory of Language and Culture. Chicago, IL: University of Chicago Press.

[B37] Goldberg A. E. (2003). Constructions: a new theoretical approach to language. Trends Cogn. Sci. 7, 219–224. 10.1016/S1364-6613(03)00080-9 12757824

[B38] Goschler J. Stefanowitsch A. (Eds.). (2013). Variation and Change in the Encoding of Motion Events. Amsterdam: John Benjamins.

[B39] Gries S. (2012). Frequencies, probabilities, and association measures in usage-/exemplar-based linguistics: some necessary clarifications. Stud. Lang. 36, 477–510. 10.1075/sl.36.3.02gri

[B40] Gries S. Ellis N. C. (2015). Statistical measures for usage-based linguistics. Lang. Learn. 65, 228–255. 10.1111/lang.12119

[B41] Gries S. T. (2015). The most under-used statistical method in corpus linguistics: multi-level (and mixed-effects) models. Corpora 10, 95–125. 10.3366/cor.2015.0068

[B42] Gullberg M. Burenhult N. (2012). Probing the linguistic encoding of placement and removal events in Swedish, in Events of Putting and Taking: A Crosslinguistic Perspective, eds Kopecka A. Narasimhan B. (Amsterdam: John Benjamins), 167–182. 10.1075/tsl.100.12gul

[B43] Haxby J. V. Connolly A. C. Guntupalli J. S. (2014). Decoding neural representational spaces using multivariate pattern analysis. Annu. Rev. Neurosci. 37, 435–456. 10.1146/annurev-neuro-062012-170325 25002277

[B44] Heidlmayr K. Weber K. Takashima A. Hagoort P. (2020). No title, no theme: the joined neural space between speakers and listeners during production and comprehension of multi-sentence discourse. Cortex 130, 111–126. 10.1016/j.cortex.2020.04.035 32652339

[B45] Hendriks H. Hickmann M. Demagny A. C. (2008). How adult English learners of French express caused motion: a comparison with English and French natives. Acquisit. Interact. Lang. Étrang. 27, 15–41. 10.4000/aile.3973

[B46] Hickmann M. Hendriks H. (2010). Typological constraints on the acquisition of spatial language in French and English. Cogn. Linguist. 21, 189–215. 10.1515/COGL.2010.007

[B47] Hijazo-Gascón A. Ibarretxe-Antuñano I. (2013). Las lenguas románicas y la tipología de los eventos de movimiento. Roman. Forsch. 125, 467–494. 10.3196/003581213808754483

[B48] Hoffmann T. Horsch J. Brunner T. (2019). The more data, the better: a usage-based account of the English comparative correlative construction. Cogn. Linguist. 30, 1–36. 10.1515/cog-2018-0036

[B49] Hwang H. Kaiser E. (2014). Having a syntactic choice is not always better: the effects of syntactic flexibility on Korean production. Lang. Cogn. Neurosci. 29, 1115–1131. 10.1080/23273798.2013.875212

[B50] Iacobini C. Masini F. (2006). The emergence of verb-particle constructions in Italian: locative and actional meanings. Morphology 16, 155–188. 10.1007/s11525-006-9101-7

[B51] Iacobini C. Vergaro C. (2014). The role of inference in motion event encoding/decoding: a cross-linguistic inquiry into English and Italian. Ling. Ling. 13, 211–240. 10.1418/78408

[B52] Ibarretxe-Antuñano I. (2009). Path salience in motion events, in Crosslinguistic Approaches to the Psychology of Language: Research in the Tradition of Dan Isaac Slobin, eds Guo J. Lieven E. Budwig N. Ervin-Tripp S. Nakamura K. Ösçaliskan S. (New York, NY: Routledge), 403–414.

[B53] Kapatsinski V. (2014). What is grammar like? A usage-based constructionist perspective. in Linguistic Issues in Language Technology—Theoretical and Computational Morphology: New Trends and Synergies (Vol. 11). CSLI Publications. Available online at: https://www.aclweb.org/anthology/2014.lilt-11.2

[B54] Kay P. (1996). Intra-speaker relativity, in Rethinking Linguistic Relativity, Volume 17 of Studies in the Social and Cultural Foundations of Language, eds Gumperz J. J. Levinson S. C. (Cambridge: Cambridge University Press), 97–114.

[B55] Kersten A. W. Meissner C. A. Lechuga J. Schwartz B. L. Albrechtsen J. S. Iglesias A. (2010). English speakers attend more strongly than Spanish speakers to manner of motion when classifying novel objects and events. J. Exp. Psychol. Gen. 139, 638–653. 10.1037/a0020507 20853990

[B56] Kopecka A. (2006). The semantic structure of motion verbs in French: typological perspectives, in Space in Languages: Linguistic Systems and Cognitive Categories, eds Hickmann M. Robert S. (Amsterdam: John Benjamins), 83–101. 10.1075/tsl.66.06kop

[B57] Kopecka A. (2009). L'expression du déplacement en français: L'interaction des facteurs sémantiques, aspectuels et pragmatiques dans la construction du sens spatial. Langages 173, 54–75. 10.3917/lang.173.0054

[B58] Kruschke J. K. Liddell T. M. (2018). The Bayesian new statistics: hypothesis testing, estimation, meta-analysis, and power analysis from a Bayesian perspective. Psychon. Bull. Rev. 25, 178–206. 10.3758/s13423-016-1221-4 28176294

[B59] Langacker R. W. (1999). Grammar and conceptualization. Berlin: De Gruyter.

[B60] Levelt W. J. M. (1989). Speaking: From Intention to Articulation. Number 99-0886244-8 in ACL-MIT Press Series in Natural Language Processing. Cambridge, MA: MIT Press.

[B61] Levinson S. C. (2003). Space in Language and Cognition: Explorations in Cognitive Diversity. Cambridge: Cambridge University Press.

[B62] Loucks J. Pederson E. (2011). Linguistic and non-linguistic categorization of complex motion events, in Event Representation in Language and Cognition, eds Bohnemeyer J. Pederson E. (Cambridge: Cambridge University Press), 108–133. 10.1017/CBO9780511782039.006

[B63] Lucy J. A. (1992). Language Diversity and Thought: A Reformulation of the Linguistic Relativity Hypothesis. Cambridge: Cambridge University Press.

[B64] Majid A. Roberts S. G. Cilissen L. Emmorey K. Nicodemus B. O'Grady L.. (2018). Differential coding of perception in the world's languages. Proc. Natl. Acad. Sci. U.S.A. 115, 11369–11376. 10.1073/pnas.1720419115 30397135 PMC6233065

[B65] Martínez Vázquez M. (2015). Satellite-framed patterns in Romance languages. Lang. Contrast 15, 181–207. 10.1075/lic.15.2.02mar

[B66] Matsumoto Y. (2003). Typologies of lexicalization patterns and event integration: clarifications and reformulations, in Empirical and Theoretical Investigations Into Language: A Festschrift for Masaru Kajita, ed Chiba S. (Tokyo: Kaitakusha), 403–418.

[B67] Montemurro M. A. Zanette D. H. (2011). Universal entropy of word ordering across linguistic families. PLoS ONE 6:e19875. 10.1371/journal.pone.0019875 21603637 PMC3094390

[B68] Montero-Melis G. Buz E. Jaeger T. F. (2016). Does syntactic flexibility in production facilitate or inhibit planning? in CUNY Conference on Human Sentence Processing (Gainesville, FL).

[B69] Montero-Melis G. Bylund E. (2017). Getting the ball rolling: the cross-linguistic conceptualization of caused motion. Lang. Cogn. 9, 446–472. 10.1017/langcog.2016.22

[B70] Montero-Melis G. Eisenbeiss S. Narasimhan B. Ibarretxe-Antuñano I. Kita S. Kopecka A.. (2017). Satellite- vs. verb-framing underpredicts nonverbal motion categorization: insights from a large language sample and simulations. Cogn. Semant. 3, 36–61. 10.1163/23526416-00301002

[B71] Naigles L. R. Eisenberg A. R. Kako E. T. Highter M. McGraw N. (1998). Speaking of motion: verb use in English and Spanish. Lang. Cogn. Process. 13, 521–549. 10.1080/016909698386429

[B72] Nikitina T. (2008). Pragmatic factors and variation in the expression of spatial goals: the case of into vs. in, in Syntax and Semantics of Spatial P, eds Asbury A. Dotlacil J. Gehrke B. Nouwen R. (Amsterdam: John Benjamins), 175–195.

[B73] Oxford University Press University of Cambridge, and Association of Language Testers in Europe. (2001). Quick Placement Test: Paper and Pen Test. Oxford: Oxford University Press.

[B74] Papafragou A. Massey C. Gleitman L. (2002). Shake, rattle, ‘n' roll: the representation of motion in language and cognition. Cognition 84, 189–219. 10.1016/S0010-0277(02)00046-X 12175572

[B75] Papafragou A. Massey C. Gleitman L. (2006). When English proposes what Greek presupposes: the cross-linguistic encoding of motion events. Cognition 98, B75–B87. 10.1016/j.cognition.2005.05.005 16043167

[B76] Papafragou A. Selimis S. (2010). Event categorisation and language: a cross-linguistic study of motion. Lang. Cogn. Process. 25, 224–260. 10.1080/01690960903017000

[B77] Ragnarsdóttir H. Strömqvist S. (2004). Time, space, and manner in Swedish and Icelandic: narrative construction in two closely related languages, in Relating Events in Narrative. Vol. 2, Typological and Contextual Perspectives, eds Strömqvist S. Verhoeven L. (Mahwah, NJ: Lawrence Erlbaum), 113–141.

[B78] Reines M. F. Prinz J. (2009). Reviving whorf: the return of linguistic relativity. Philos. Compass 4, 1022–1032. 10.1111/j.1747-9991.2009.00260.x

[B79] Schielzeth H. Nakagawa S. (2013). Nested by design: model fitting and interpretation in a mixed model era. Methods Ecol. Evol. 4, 14–24. 10.1111/j.2041-210x.2012.00251.x

[B80] Schmid H. J. (2007). Entrenchment, salience, and basic levels, in The Oxford Handbook of Cognitive Linguistics, eds Geeraerts D. Cuyckens H. (Oxford; New York, NY: Oxford University Press), 117–138.

[B81] Schmid H. J. (2017). Linguistic entrenchment and its psychological foundations, in Entrenchment and the Psychology of Language Learning: How We Reorganize and Adapt Linguistic Knowledge, ed Schmid H. J. (Washington, DC: American Psychological Association), 435–452. 10.1037/15969-020

[B82] Slobin D. I. (1996). From “thought and language” to “thinking for speaking”, in Rethinking Linguistic Relativity, eds Gumperz J. J. Levinson S. C. (Cambridge: Cambridge University Press), 70–96.

[B83] Slobin D. I. (2004). The many ways to search for a frog: linguistic typology and the expression of motion events, in Relating Events in Narrative: Typological and Contextual Perspectives, eds Strömqvist S. Verhoeven L. (Mahwah, NJ: Erlbaum), 219–257.

[B84] Slobin D. I. (2005). Linguistic representation of motion events: what is signifier and what is signified? in Outside-In, Inside-Out, Volume 4 of Iconicity in Language and Literature, eds Maeder C. Fischer O. Herlofsky W. J. (Amsterdam: John Benjamins), 307–322. 10.1075/ill.4.22slo

[B85] Slobin D. I. Bowerman M. Brown P. Eisenbeiss S. Narasimhan B. (2011). Putting things in places: developmental consequences of linguistic typology, in Event Representation in Language and Cognition, eds Bohnemeyer J. Pederson E. (Cambridge: Cambridge University Press), 134–165. 10.1017/CBO9780511782039.007

[B86] Slobin D. I. Hoiting N. (1994). Reference to movement in spoken and signed languages: typological considerations, in Proceedings of the Twentieth Annual Meeting of the Berkeley Linguistics Society (Berkeley, CA: Berkeley Linguisitcs Society), 487–505. 10.3765/bls.v20i1.1466

[B87] Slobin D. I. Ibarretxe-Antuñano I. Kopecka A. Majid A. (2014). Manners of human gait: a crosslinguistic event-naming study. Cogn. Linguist. 25, 701–741. 10.1515/cog-2014-0061

[B88] Swoyer C. (2011). How does language affect thought? in Language and Bilingual Cognition, eds Cook V. Bassetti B. (New York, NY: Psychology Press), 23–42.

[B89] Talmy L. (1991). Path to realization: a typology of event conflation, in Proceedings of the Seventeenth Annual Meeting of the BLS (Berkeley, CA: Berkeley Linguisitcs Society), 480–519. 10.3765/bls.v17i0.1620

[B90] Talmy L. (2000). Toward a Cognitive Semantics: Typology and Process in Concept Structuring. Cambridge, MA: MIT Press.

[B91] Verhagen V. Mos M. (2016). Stability of familiarity judgments: individual variation and the invariant bigger picture. Cogn. Linguist. 27, 307–344. 10.1515/cog-2015-0063

[B92] Verhagen V. Mos M. Schilperoord J. Backus A. (2020). Variation is information: analyses of variation across items, participants, time, and methods in metalinguistic judgment data. Linguistics 58, 37–81. 10.1515/ling-2018-0036

[B93] Verkerk A. (2014). Where Alice fell into: motion events from a parallel corpus, in Aggregating Dialectology, Typology, and Register Analysis: Linguistic Variation in Text and Speech, eds Szmrecsanyi B. Wälchli B. (Berlin: De Gruyter), 324–354.

[B94] von Stutterheim C. Nüse R. (2003). Processes of conceptualization in language production: language-specific perspectives and event construal. Linguistics 41, 851–881. 10.1515/ling.2003.028

[B95] Whorf B. L. (1956). Language, Thought, and Reality. Selected Writings of Benjamin Lee Whorf. Cambridge, MA: MIT Press.

[B96] Wolff P. Holmes K. J. (2011). Linguistic relativity. Wiley Interdiscipl. Rev. Cogn. Sci. 2, 253–265. 10.1002/wcs.104 26302074

[B97] Zlatev J. Yangklang P. (2004). A third way to travel: the place of Thai in motion event typology, in Relating Events in Narrative: Typological and Contextual Perspectives, eds Strömqvist S. Verhoeven L. (Mahwah, NJ: Erlbaum), 159–190.

[B98] Zwaan R. A. (1999). Situation models: the mental leap into imagined worlds. Curr. Direct. Psychol. Sci. 8, 15–18. 10.1111/1467-8721.00004

[B99] Zwaan R. A. (2016). Situation models, mental simulations, and abstract concepts in discourse comprehension. Psychon. Bull. Rev. 23, 1028–1034. 10.3758/s13423-015-0864-x 26088667 PMC4974264

